# Measurement of the extinction coefficients of magnetic fluids

**DOI:** 10.1186/1556-276X-6-237

**Published:** 2011-03-18

**Authors:** Xiaopeng Fang, Yimin Xuan, Qiang Li

**Affiliations:** 1 School of Power Engineering, Nanjing University of Science and Technology, Nanjing, 210094, China

## Abstract

A novel spectral transmittance approach for measuring the extinction coefficient of magnetic fluids is proposed. The measuring principle and accuracy of the approach are analysed. Experiments are conducted to measure the extinction coefficient of magnetic fluids with different particle volume fractions. The relative uncertainty of experimental data is less than 1.8%. The experimental results indicate that the extinction coefficient of magnetic fluids increases with increase of the volume fraction of suspended magnetic nanoparticles and the optical properties of the particle material have a significant effect on the extinction coefficient of the magnetic fluids.

## Introduction

Magnetic fluid is a kind of colloidal suspension containing surfactant-coated magnetic nanoparticles dispersed in carrier liquids such as water and oil. Since the emergence of magnetic fluid in the 1960s, researchers have conducted many investigations on the characters of magnetic fluids. When light is incident into the magnetic fluid, the magnetic nanoparticles suspended in the base fluid will interact with the incident light and affect the propagation of the incident light. Magnetic fluid presents some special optical characters such as birefringence [1-5] and magnetochromatics [6,7]. Recently, the remarkable magneto-optical characters of magnetic fluids have attracted enormous attention of researchers who tried to apply magnetic fluids to optical devices such as fabricating optical switches [8], tunable filters [9], magnetic-field sensors [10] and tunable optical grating [11,12]. Although the optical characters of the magnetic fluids have been studied for decades and novel phenomena have been discovered, there are few reports on the optical constants of magnetic fluids. Yang et a1. [13] measured the magnetic field-dependent refractive index of magnetic fluid films by total reflection technique. Pu et a1. [14] designed an experimental system for measuring the refractive index of magnetic fluid and studied the effect of particle volume fraction and temperature on the refractive index. Both of these investigations had the limitation of having one fixed wavelength, besides the experimental systems employed being complicated and expensive. So far, few investigations on the extinction coefficient of magnetic fluid have been reported. To promote the applications of magnetic fluids in different fields such as thermal and optical engineering, many more efforts toward further investigation of optical constants of magnetic fluids are needed. The purpose of this letter is to propose a simple method for measuring the extinction coefficient of magnetic fluids.

## Experimental principle and apparatus

With the development of the manufacturing technique, the spectral transmittance of a translucent film can be accurately measured by a spectrophotometer. Based on the film transmission principle, the method for measuring the extinction coefficient of magnetic fluids can be established. The procedure of our method is as follows: first, two magnetic fluid films with different thicknesses are prepared. The spectral transmittance of these films can be accurately measured using a spectrophotometer. Then, based on the film transmission principle, two transmittivity equations of the magnetic fluid films with regard to the optical constant of the magnetic fluid are established. Finally, by solving the two equations, the extinction coefficient of the magnetic fluid can be obtained.

For fabricating a magnetic fluid film, two JGS3 glasses and a polyester film are needed. The glass has 50-mm width, 50-mm length and 1-mm thickness. The polyester film has a 30-mm-diameter circular hole in the central section. The polyester film is sandwiched in between the two glasses, and the magnetic fluid is injected into the circular hole. Then, the three-layer film is clamped by an attachment. We can obtain magnetic fluid film of different thicknesses by choosing polyester films of different thicknesses.

**Figure 1:**
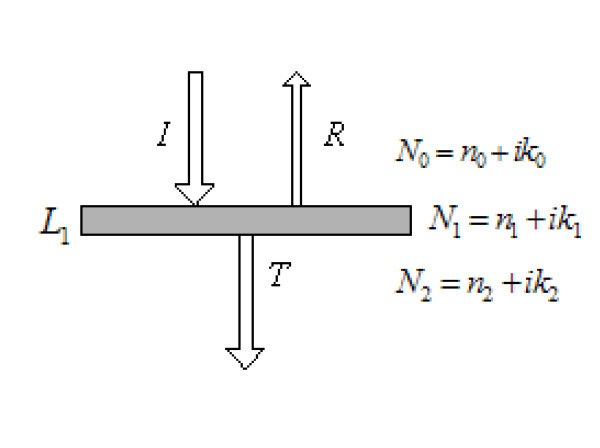
**Reflection and transmission by a glass slab**.

For a light beam with normal incidence on a slab (as shown in Figure 1), the reflectivity *R *and the transmittivity *T *of the slab are(1)(2)(3)(4)

where *n *is the refractive index, *k *denotes the extinction coefficient, *L *denotes the thickness, λ denotes wavelength, and subscripts *i *and *j *represent the different media.

**Figure 2:**
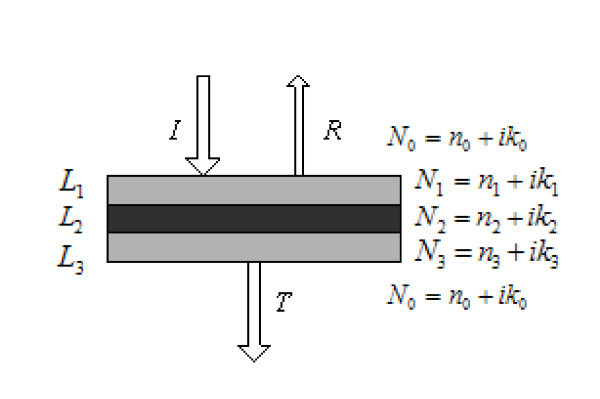
**Reflection and transmission by magnetic fluid film**.

As shown in Figure 2, the magnetic fluid film is a three-layered structure. Its effective transmittivity *T*_eff _can be obtained using the effective interface law [15].(5)(6)(7)(8)(9)(10)

## Precision analysis

**Figure 3:**
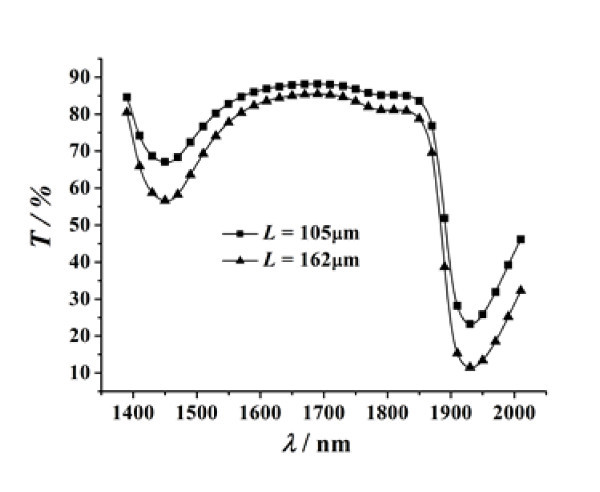
**Spectral transmittance of water films with two different thicknesses**.

**Figure 4:**
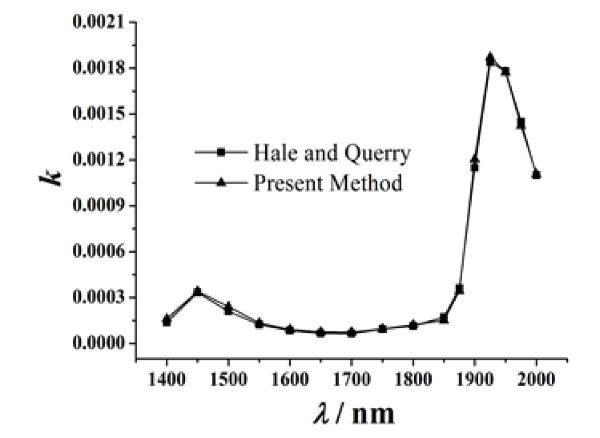
**Comparison of the result measured by the present approach and that of Hale and Querry's method for extinction coefficients of water at 25°C**.

In order to validate the accuracy of this method, the extinction coefficient of water was measured at 25°C. First, two water films of different thicknesses were fabricated. Then, the transmittivity values of the two films were measured using a Lambda 950 spectrophotometer, and the results are illustrated in Figure 3. Consequently, the two transmittivity equations of the water films could be established according to Equations 1-10. These equations are solved using Levanberg-Marquardt arithmetic and global optimization method, and the extinction coefficients of the water film are obtained. As shown in Figure 4, the results obtained from the present method are in good concurrence with those obtained from Hale and Querry's approach [16].

The data uncertainty results mainly from the measuring errors of structure parameters and transmittivity of the glass. To estimate the data uncertainty, *L*_w1 _is used for denoting the thickness of the thin water film, *L*_w2 _for the thickness of the thick water film, *T*_eff 1 _for the transmittivity of the thin water film and *T*_eff 2 _for the transmittivity of the thick water film. A micrometer with the measurement uncertainty of 1 μm is used. For the thin water film, the thicknesses of the two glasses *L*_1 _and *L*_2 _and the whole thickness of the three-layer film *L *are, respectively, measured using the micrometer. Thus, the thickness of the thin water film is obtained from *L*_*w1 *_= *L *- *L*_1 _- *L*_2_, and the measurement uncertainty of the film thickness from *δL *= *δL*_1 _= *δL*_2 _= 1 μm. Therefore, the uncertainty of *L*_w1 _and *L*_w2 _is determined as  = 1.732 μm.

The relative uncertainty of transmittivity of a Lambda 950 spectrophotometer is given to be 0.1%. Given the operational error during the measurement, the relative uncertainty of 0.5% is assumed for the transmittivity, i.e., the uncertainty of *T*_eff 1 _is written as δ*T*_eff1 _= 0.005*T*_eff1_, and the uncertainty of *T*_eff 2 _as δ*T*_eff2 _= 0.005*T*_eff2_. Finally, the uncertainty of the extinction coefficient can be theoretically estimated as(11)

From the above expression, one can estimate the relative uncertainty of the extinction coefficient of sample films. The relative uncertainties of the measured extinction coefficients of water are shown in Table 1. Figure 4 and Table 1 illustrate that the extinction coefficient and the relative uncertainty vary at different wavelengths for water. For λ = 1700 nm, the measured extinction coefficient of water is *k *= 7.4 × 10^-5^, and the relative uncertainty is 16.53%. For λ = 1925 nm, the measured extinction coefficient of water film is *k *= 1.87 × 10^-3^, and the relative uncertainty is 4.47%. The relative uncertainty of the experimental data decreases with the increase of the extinction coefficient. It indicates that the method used in this study is precise and suitable for measuring fluids with a large extinction coefficient.

**Table 1:** The relative uncertainty of the measured extinction coefficient of water at different wavelengths

λ (nm)	1400	1450	1500	1550	1600	1650	1700	1750
Relative uncertainty (%)	9.57	5.92	7.60	10.94	13.43	15.72	16.53	14.31

λ (nm)	1800	1850	1875	1900	1925	1950	1975	2000

Relative uncertainty (%)	12.17	10.93	4.67	4.67	4.47	4.49	4.59	4.74

## Results and discussion

**Figure 5:**
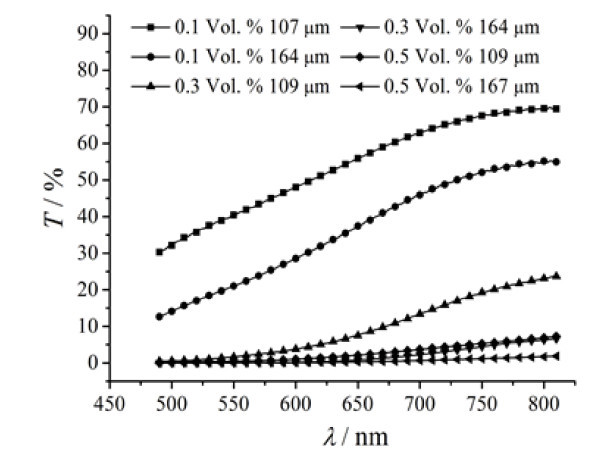
**Spectral transmittance of magnetic fluid films with different thicknesses and various particle volume fractions**.

The samples of water-based Fe_3_O_4 _magnetic fluid with three different particle volume fractions of 0.1, 0.3 and 0.5% were prepared by the chemical coprecipitation method. For each particle volume fraction, two magnetic fluid films with different thicknesses were prepared. At the ambient temperature of 25°C, the spectral transmittance of the magnetic fluid films was measured using a Lambda 950 spectrophotometer, and the experimental data are illustrated in Figure 5.

**Figure 6:**
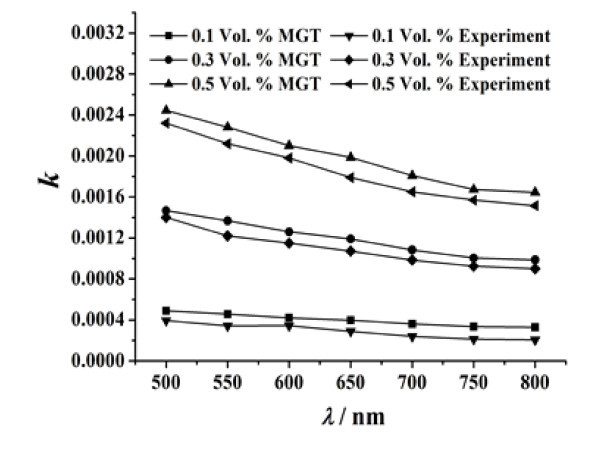
**Comparison of the result measured by the experiment and that predicted by the MGT for extinction coefficients of magnetic fluids with different particle volume fractions**.

Based on the above mentioned procedure, the extinction coefficients of the magnetic fluids were calculated. The calculated extinction coefficients of magnetic fluids with different particle volume fractions are shown in Figure 6. Then, the relative uncertainties of the calculated extinction coefficients were estimated, and the results are shown in Table 2. Obviously, the extinction coefficient of the magnetic fluid and the data uncertainty vary with the change of particle volume fraction and the wavelength of the incident light. The largest relative uncertainty of experimental data is 1.73% at λ = 550 nm for 0.1% particle volume fraction.

**Table 2:** The relative uncertainty of the measured extinction coefficient of magnetic fluids with different particle volume fractions at different wavelengths

ϕ	λ (nm)						
	
	500	550	600	650	700	750	800
0.1%	1.67%	1.73%	1.68%	1.69%	1.67%	1.69%	1.72%

0.3%	1.59%	1.60%	1.60%	1.61%	1.63%	1.65%	1.66%

0.5%	1.58%	1.59%	1.60%	1.60%	1.61%	1.62%	1.63%

Effective medium theory (EMT) is a powerful method to predict the optical properties of composite materials. One of the most popular EMT is the Maxwell--Garnett theory (MGT) [17]. In this letter, the particle volume fraction of magnetic fluid is low, and the particle size is much smaller than the incident wavelength. Hence, here the MGT is introduced to predict the extinction coefficient of magnetic fluid. The MGT formula for the dielectric function of magnetic fluid is expressed as(12)

where ε_r,w _is the dielectric function of water, ε_r,p _is the dielectric function of Fe_3_O_4 _and ϕ is the particle volume fraction.

The relationship between the complex refractive index NM = *n + ik *and the dielectric function  is [18](13)(14)(15)(16)

**Figure 7:**
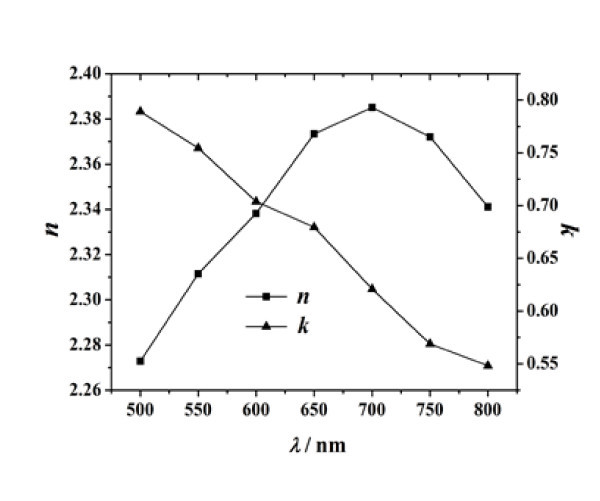
**Refractive index *n *and extinction coefficient *k *of Fe_3_O_4_**.

The dielectric function of Fe_3_O_4 _can be obtained from the research of Schlegel [19]. Using Equations 15 and 16, the refractive index and extinction coefficient of Fe_3_O_4 _were calculated, and the results are shown in Figure 7.

Since the complex refractive index of water can be obtained from Ref. [16], the dielectric function of water was calculated using Equations 13 and 14. So far, the dielectric functions of water and Fe_3_O_4 _are both available. Based on the MGT formula, the dielectric function of magnetic fluid is calculated. Then, according to Equations 15 and 16, the extinction coefficients of magnetic fluid were predicted. The predicted extinction coefficients of magnetic fluids with different particle volume fractions are shown in Figure 6. It can be seen from Figure 6 that the result of the MGT is a little higher than that of the experiment. This is because the MGT is a reduced model for the optical properties of magnetic fluid. The influence of both the Brownian motion of magnetic particles and the aggregation of magnetic particles on the optical properties of magnetic fluid is not considered in the MGT. Nevertheless, these two factors are not dominant for the optical feature of the magnetic fluid. Hence, the variation trend of the MGT result concurs with that of the experimental result.

From the curves in Figure 6, one learns that the extinction coefficient of magnetic fluid increases with the increase in the particle volume fraction. When the incident wavelength is 800 nm, the extinction coefficient of water is *k *= 1.25 × 10^-7 ^from Ref. [16] and the extinction coefficient of magnetic fluid of 0.1% particle volume fraction (experimental result) is *k *= 2.06 × 10^-4^. The latter is 1647 times than the former. This is because the size of suspended magnetic nanoparticles is much smaller than the incident wavelength. According to the Mie's scattering theory, the absorption of the incident light is the main optical character of these magnetic nanoparticles. The presence of the magnetic nanoparticles in water greatly enhances the total absorption of light. With an increase of the particle volume fraction, the number of particles in the unit volume increases, and the total absorption of light further increases.

From Figure 6, it can be seen that in the continuous wavelength range of 500-800 nm, the extinction coefficients of magnetic fluids decrease with the increase of the incident wavelength. This is because the spectral features of the suspended magnetic nanoparticles are dominant for the optical feature of the magnetic fluid film. Since the extinction coefficient of Fe_3_O_4 _particles decreases with the increase of the wavelength (see Figure 7) and the scattering function of these nanoparticles is extremely weak, the absorption of light by the nanoparticles decreases with an increase in the wavelength. In other words, the above phenomenon further indicates that the optical properties of the nanoparticles play a significant role in affecting the optical properties of magnetic fluids.

## Conclusions

In this study, a simple spectral transmittance method was proposed for measuring the extinction coefficient of magnetic fluids. The extinction coefficients of magnetic fluids with different particle volume fractions in the continuous wavelength range of 500-800 nm were measured. The experimental data have revealed that the extinction coefficient of magnetic fluids increases with increase of the particle volume fraction, and the optical properties of the particle material have a significant effect on the extinction coefficient of the magnetic fluids. It is feasible to control the optical character of the magnetic fluids by changing the optical properties of the nanoparticles.

## Abbreviations

EMT: Effective medium theory; MGT: Maxwell-Garnett theory.

## Competing interests

The authors declare that they have no competing interests.

## Authors' contributions

The experimental measurement and the theoretical calculation of this study were mainly done by the first author, XF. YX contributed to both the experimental and theoretical work and gave some useful suggestions to XF, which helped him finish the work. QL contributed to the experimental work and gave XF some help. This article was written by XF and revised by YX.
